# Technologies for frailty, comorbidity, and multimorbidity in older adults: a systematic review of research designs

**DOI:** 10.1186/s12874-023-01971-z

**Published:** 2023-07-11

**Authors:** Alessia Gallucci, Pietro D. Trimarchi, Cosimo Tuena, Silvia Cavedoni, Elisa Pedroli, Francesca Romana Greco, Antonio Greco, Carlo Abbate, Fabrizia Lattanzio, Marco Stramba-Badiale, Fabrizio Giunco

**Affiliations:** 1 IRCCS Fondazione Don Carlo Gnocchi, Milan, Italy; 2 Applied Technology for Neuro‑Psychology Lab, IRCCS Istituto Auxologico Italiano, Milan, Italy; 3 Faculty of Psychology, University of eCampus, Novedrate, Italy; 4 Geriatric Unit, Department of Medical Sciences, IRCCS ‘‘Casa Sollievo della Sofferenza’’, San Giovanni Rotondo, Italy; 5 Scientific Direction, IRCCS INRCA, Ancona, Italy; 6 Department of Geriatrics and Cardiovascular Medicine, IRCCS Istituto Auxologico Italiano, Milan, Italy

**Keywords:** Frailty, Health technology, Rehabilitation, Aging, Research methodology

## Abstract

**Background:**

Frailty, neurodegeneration and geriatric syndromes cause a significant impact at the clinical, social, and economic level, mainly in the context of the aging world. Recently, Information and Communication Technologies (ICTs), virtual reality tools, and machine learning models have been increasingly applied to the care of older patients to improve diagnosis, prognosis, and interventions. However, so far, the methodological limitations of studies in this field have prevented to generalize data to real-word. This review systematically overviews the research designs used by studies applying technologies for the assessment and treatment of aging-related syndromes in older people.

**Methods:**

Following the PRISMA guidelines, records from PubMed, EMBASE, and Web of Science were systematically screened to select original articles in which interventional or observational designs were used to study technologies’ applications in samples of frail, comorbid, or multimorbid patients.

**Results:**

Thirty-four articles met the inclusion criteria. Most of the studies used diagnostic accuracy designs to test assessment procedures or retrospective cohort designs to build predictive models. A minority were randomized or non-randomized interventional studies. Quality evaluation revealed a high risk of bias for observational studies, while a low risk of bias for interventional studies.

**Conclusions:**

The majority of the reviewed articles use an observational design mainly to study diagnostic procedures and suffer from a high risk of bias. The scarce presence of methodologically robust interventional studies may suggest that the field is in its infancy. Methodological considerations will be presented on how to standardize procedures and research quality in this field.

**Supplementary Information:**

The online version contains supplementary material available at 10.1186/s12874-023-01971-z.

## Introduction

Worldwide, life expectancy is rapidly increasing and according to the previsions, the proportion of people older than 60 years will reach 21.1% by 2050 compared to the 9.2% in 1990 and the 11.7% in 2013 (World Health Organization; https://www.who.int/news-room/fact-sheets/detail/ageing-and-health). Despite the improvement of instruments and standards of care, late life is not empty of complex chronic medical conditions that represent major problems in particular for health care systems still focusing on a disease-oriented approach [1].

Accordingly, the transition towards an aging world is boosting a gradual passage to more person-focused care models [2]. Within these models, frailty, comorbidity, and multimorbidity have recently caught the attention of scholars and clinicians, with a significant increase of publications [3], even about the application of technological tools for diagnosis and intervention of these conditions. However, recent literature reviews underlined limitations linked to methodological procedures of research conducted in this field, reducing studies’ validity and results’ generalizability [4, 5]. Indeed, most of the studies testing technology feasibility use observational designs with assessment purposes in limited and heterogeneous samples of frail, comorbid, or multimorbid older patients, while interventional studies involving groups of patients defined by clear inclusion and exclusion criteria are under-represented. These methodological difficulties may be also linked to the interchangeable modality by which frailty, comorbidity, and multimorbidity are often defined, with a lack of consensus regarding their operational translation in practice [6]. Although frequently used as synonyms, these conditions are separate clinical concepts [7] that can independently coexist or mutually interact constituting pre-disability conditions [8].

There is an agreement depicting frailty as a dynamic and multicomponential condition depending on or leading to an extreme vulnerability to stressors and reduced resiliency [9, 10]. Despite this consensus, frailty has been alternatively defined as the result of the accumulation of deficits [11] or as a clinical syndrome (i.e., Frailty Phenotype) [7], with both these definitions failing to include symptoms belonging to psychosocial and cognitive domains, that are instead captured by the more recent concept of intrinsic capacity [12, 13].

The controversy about definitions of multimorbidity and comorbidity depends on the nature, time of onset, and assessment of the diagnosed clinical diseases [14] that co-occur, not interdependently, in the case of multimorbidity or that generate combined effects in reference to an index chronic disease in the case of comorbidity [15].

The prevalence of frailty, comorbidity and multimorbidity increases with aging [16], leading to reduce life expectancy, impairments in daily living activities and postoperative complications, increased risk of mortality, and costs for public health, in terms of frequency and duration of emergency room visits and hospital admissions [17–20]. Therefore, care pathways aiming at personalizing interventions based on the needs of older patients, namely patient-centered health systems, may be crucial to cover the urgency to manage the impactful long-term consequences of these conditions [17]. Going further, these novel care approaches could benefit from the use of assistive health technology (i.e., technological solutions aiming to maintain or improve functionality, autonomy, and well-being) or medical devices (i.e., technological solutions aiming to support prevention, diagnosis, and treatment) to prioritize integration between different settings of care and care professionals, the inclusion of caregivers in the treatment programs, development of remote self-management solutions and procedures [21–23].

Accordingly, in the last years, research showed that Information and Communication Technologies (ICTs), machine learning algorithms/models [24], and virtual reality tools [25, 26] may be beneficial for older people [27, 28]. In particular, technologies demonstrated to ameliorate diagnosis, prognosis, and prevention strategies [29] as well as facilitate remote monitoring [30], continuity of care [31], access to healthcare services [32] and even patients’ independence and quality of life [33].

However, studies pointed out also barriers to the use of technology in samples of frail, comorbid, and multimorbid older people, preventing cost-effectiveness analysis and solid integration of technologies within complex assistive models. Beyond technical and economic aspects [33, 34], as already mentioned, research seems to lack rigorous methodological approaches, with a clear displacement towards assessment of frailty, comorbidity, and multimorbidity compared to clinical trials testing technologies as intervention tools in samples suffering from complex geriatric syndromes. Indeed, technologies have been mostly tested in reference to their technical aspects, whereas applications in clinical settings frequently are pilot experiences on small and mixed groups of patients, not providing analysis of patients’ needs, considerations about usability and acceptability of tested devices, exploration of characteristics of the real-world application scenarios [4, 5].

Despite these limitations, to the best of our knowledge, literature still lacks a comprehensive review of research designs, in terms of observational or interventional designs, that studies using technologies for the assessment and treatment of aging-related syndromes in older people have applied so far.

This review aims to fill this gap by systematically describing research designs and procedures currently applied to transfer laboratory results to real-world practices in order to critically appraise studies’ methodological quality based on structured criteria and present considerations on how to standardize studies’ methods and research quality in this field.

## Methods

### Literature search

Our systematic review was conducted according to the Preferred Reporting Items for Systematic Reviews and Meta-Analyses (PRISMA) guidelines [35]. PROSPERO registration number: CRD42020218053.

The selected keywords were: “frailty”, “multimorbidity”, “comorbidity”, “aging”/“elderly” that were combined, into three arms, with ICT, machine learning, and virtual reality [33, 36–39]. See Appendix A in the Supplementary material for the details of the search strategies and combinations.

The arms were searched as major topics in Pubmed, Web of Science, and Embase (Ovid), restricting the literature search to title, abstract, and keywords.

Using a web and mobile systematic review manager [40], after duplicates removal, four blinded researchers (A.G., P.D.T., C.T., S.C.) in pairs categorized the records as “included”, “excluded”, or “unsure” based on title/abstract. Then, during the full-text screening, records included or categorized as “unsure” in the first stage were reviewed. In both screening stages conflicts were resolved by consensus of the researchers of each pair and a third author was consulted if discrepancies remained. The authors of papers whose full-text was not available were contacted.

### Selection criteria

Eligible for inclusion were peer-reviewed studies published to the end of September 2020. The following hierarchy of eligibility criteria was adopted: (a) English written articles; (b) articles involving human samples; (c) peer-reviewed articles; (d) articles including frail or multimorbid or comorbid participants older than 65 years. In particular, we considered records in which frailty or multimorbidity or comorbidity were the main outcomes of the technology-based assessment or intervention, while we excluded studies where these conditions were simply assessed to describe participants but were not part of the studies’ aims; (e) articles applying technologies with clear diagnostic or intervention purposes. Therefore, we excluded records including technologies only to assess their usability or acceptability. Moreover, we included papers on telerobotics devices, while robotics used in surgical settings were excluded; (f) original peer-reviewed articles with interventional or observational study designs according to types proposed in [41], while narrative or systematic reviews, meta-analyses, case-reports, abstracts, conference proceedings and study protocols were excluded.

### Data extraction and synthesis

Data from each of the included studies were collected by one of the authors in each pair by using a specific form. Data were checked for accuracy and completeness by the other pair’s member and discrepancies were solved by consensus and/or by a third author if needed (see Table 1).

**Table 1:** Characteristics of the included studies

Paper	Sample (N)	Age	Condition	Condition assessment or criteria	Outcome variable	Adopted technology	Research methodology	General aim	Specific aim
Alqahtani et al., 2017 [55]	N = 29	mean 87, sd 6	Frailty	Fried criteria	Upright balance, lower extremity muscle strength	Balance accelerometer; uni-axial load cell	Observational (Diagnostic accuracy study design)	Assessment	Validation of inexpensive measurements of strength and balance
Ambagtsheer et al., 2020 [56]	N = 592	median 88, (IQR 9.0)	Frailty	Electronic Frailty Index	Clegg’s 36-items: activity limitation, chronic disease, falls, social isolation, cognition, mobility, polypharmacy, sleep quality and weight loss.	Machine Learning: K-Nearest Neighbours, Decision Tree, Support Vector Machines	Observational (Diagnostic accuracy study design)	Assessment	Identifying frailty from administrative records
Boumans et al., 2019 [62]	N = 42	mean 77.1, sd 5.7	Frailty	Frailty Index	Time for completion of the questionnaires/robot–patient and nurse–patient interactions; percentage of robot–patient interactions completed autonomously	Social robot	Observational (Diagnostic accuracy study design)	Assessment	Effectiveness and acceptability of robot assistant assessment
Camicioli et al., 2015 [63]	N = 72	mean 74.97, sd 1.44	Frailty	Fried criteria	Handwriting parameters: velocity, pressure, pauses.	Writing tablet with an instrumented pen for quantifying three-dimensional aspects of copying	Observational (Diagnostic accuracy study design)	Assessment	Studying relation between handwriting measures and frailty
Dupuy et al., 2017 [45]	N = 32	mean 81.63, sd 1.57	Frailty	Fried criteria	Everyday activities; safety; social participation; interaction support; functional status; caregiver burden	Assisted-living platform: a set of wireless sensors and two touchscreen tablets	Interventional (Randomized controlled trial)	Intervention	Enhancing ADL authonomy, safety and sociality
Galan-Mercant & Cuesta-Vargas, 2013 [64]	N = 30	mean 76.98, sd 4.85	Frailty	Fried criteria	Variability of the three-axes accelerations, angular velocity, and displacement of the trunk during the Si-St and St-Si transitions	iPhone 4 accelerometer	Observational (Diagnostic accuracy study design)	Assessment	Detecting frailty from Sit-to-Stand and Stand-to-Sit transition measures
Galan-Mercant & Cuesta-Vargas, 2014 [65]	N = 18	mean 79.95, sd 5.37	Frailty	Fried criteria	Magnitude of accelerometry values	Trialxial gyroscope, accelerometer and a magnetometer in the iPhone 4 smartphone	Observational (Diagnostic accuracy study design)	Assessment	Improving the traditional assessment tools
Galan-Mercant & Cuesta-Vargas, 2015 [66]	N = 30	mean 76.98, sd 4.85	Frailty	Fried criteria	ETUG test: sit-to-stand, gait go, turning, gait come, turn-to-stand-to-sit	Tri-axial gyroscope, an accelerometer and a magnetometer in the iPhone 4.	Observational (Diagnostic accuracy study design)	Assessment	Using intertial sensors embedded in a smartphone to measure kinematic variables in frail elderly
Garcia-Moreno et al., 2020 [67]	N = 79	mean 75	Frailty	Fried criteria	Fried criteria: “non-frail” 0 criteria, “pre-frail” 2 criteria, “frail” ≥3 criteria	Samsung Gear S3 wearable sensors; Microservices System Architecture; Frailty Status App; Cloud Server; Machine Learning algorithms	Observational (Diagnostic accuracy study design)	Assessment	To assess frailty status during the performance of IADLs
Gianaria et al., 2016 [68]	N = 30	mean 75.6, sd 7.5	Frailty	Tillburg Frailty Indicator	Walking time/speed, covered distance, swing time, double support time, balance during walking, torso inclination angle	Microsoft Kinect sensors with skeleton tracking feature	Observational (Diagnostic accuracy study design)	Assessment	Detecting frailty from gait and posture features
Golkap et al., 2018 [69]	N = 36	mean 82, sd 10	Frailty	Edmonton Frail scale	Arterial hemoglobin oxygen saturation; movement in a location; bed or chair occupancy	Home Monitoring Platform: sensors to acquire patient’s habits/clinical data; home gateway, a remote server to store patient data; clinician portal to view and manage patient data	Observational (Diagnostic accuracy study design)	Assessment	Studying an integrated care system to support independent living of frailty
Graňa et al., 2020 [76]	N = 645	mean 84.2 sd 6.76	Frailty	Fried criteria	Readmissions rates	Machine Learning - Linear discrimination analysis, Support vector machines, Multilayer perceptrons, K nearest neighbors, Random forests	Observational (Retrospective cohort study design - Predictive model)	Prediction	Studying frailty as a predictor of hospital readmissions
Hassler et al., 2019 [72]	N = 474	≥ 65	Frailty	Fried criteria	≥ 3 Fried criteria	Machine Learning – naïve Bayes classifier (NB), CART algorithm tree and bagging CART, C5.0 algorithm, Random Forest analysis, SVM, LDA	Observational (Retrospective cohort study design - Predictive model)	Prediction	Finding predictive factors for frailty
Held et al., 2017 [77]	N = 1686	≥ 70 years	Geriatric syndromes (Frailty; Cognitive impairment; Falls; Incontinence)	Fried criteria; Clinical assessment for cognition; The International Consultation of Incontinence Questionnaire; ≥ 2 falls in 12 months	Frequency of medication combinations	Machine Learning - Association Rule, Frequent-Set analysis	Observational (Cross-sectional study design)	Prevalence	Detect patterns of medication combinations according to geriatric syndrome status
Kubicki et al., 2014 [49]	N = 46	mean 81.87, sd 5.9	Frailty	Fried criteria	Postural control, rapid arm movement	2D virtual reality-based program of motor telerehabilitation	Interventional (Randomized controlled trial)	Intervention	Enhancing postural control and balance
Kubicki, 2014 [57]	N = 37	mean 82.25, sd 6.01	Frailty	Fried criteria	Gait speed; hand maximal velocity; timed up and go	Semi-immersive virtual reality with active motion-capture system based onvision technology	Observational (Diagnostic accuracy study design)	Assessment	improving detection of motor control efficiency
Lee et al., 2019 [51]	N = 65	≥ 65	Frailty	Custom questionnaire (based on Study of Osteoporotic Fractures index)	Health status, exercise, frailty, handgrip, body mass	Smart phone learning and balance/flexibility exercise	Interventional (Non-randomized trial)	Intervention	Reducing frailty
Martin-Lesende et al., 2016 [70]	N = 83	mean 81.3 (IQR: 77.1–85.4)	Multimorbidity	Presence of heart failure and/or chronic lung disease; ≥ 2 admission to hospital in the previous year	Mortality rate	Telemonitoring	Observational (Retrospective cohort study design)	Mortality rate	To assess mortality according to multimorbidity and telemonitoring status
Mateo-Abad et al., 2020 [52]	N = 856	mean 77.6, sd 7.7	Multimorbidity	CIRS	Use of health care services, clinical control of the examined conditions, physical functional status, patient ´s satisfaction.	ICT-based platforms	Interventional (Non-randomized trial)	Intervention	Impact of an integrated care program on health resources use, clinical outcomes, and functional status
Merchant et al., 2020 [71]	N = 2.589	mean 73.1, sd 6.5	Geriatric Syndromes (Frailty, Cognitive impairment, Sarcopenia, Anorexia of aging)	FRAIL questionnaire	Prevalence of frailty, sarcopenia, anorexia of aging	iPad mobile application for Rapid Geriatric Assessment	Observational (Cross-sectional study design)	Prevalence	Studying prevalence of frailty, sarcopenia and anorexia of aging
Orlandoni et al., 2016 [46]	N = 188	mean 85.47, sd 7.03	Multimorbidity	CIRS	Incidence rates of complications, outpatient hospital visits, hospitalizations	Samsung Galaxy tablet for video consultation	Interventional (Randomized controlled trial)	Intervention	Enhancing home enteral nutrition management
Ozaki et al., 2017 [54]	N = 27	mean 73, sd 6	Frailty	Fried criteria	Preferred and maximal gait speeds, tandem gait speeds, timed up-and-go test, functional reach test, functional base of support, postural stability, muscle strength of the lower extremities, grip strength	Balance exercise assist robot	Interventional (Cross-over randomized controlled trial)	Intervention	Enhancing balance and walking
Paliokas et al., 2020 [58]	N = 80	mean 78.08, sd 5.48	Frailty	Fried criteria	Errors related to the product types/number, payment errors, overall duration, selected item types/number, payment score, overall score	Non-immersive Virtual Reality Serious Game	Observational (Diagnostic accuracy study design)	Assessment	Detecting frailty from Virtual Reality Serious Game
Parvaneh et al., 2017 [59]	N = 120	mean 78, sd 8	Frailty	Fried criteria	Daily postural transition	Unobtrusive shirt-embedded sensor with a three-axis accelerometer	Observational (Diagnostic accuracy study design)	Assessment	Identifying frailty from daily postural transitions
Peng et al., 2020 [73]	N = 86.133	mean 82.5	Frailty	Multimorbidity frailty index	All-cause mortality; unplanned hospitalizations; intensive care unit admissions.	Machine Learning - random forest method, Kaplan-Meier survival curve/log-rank test, Cox proportional hazard models	Observational (Retrospective cohort study design - Predictive model)	Predicition	Developing a machine learning–based multimorbidity frailty index
Persson et al., 2020 [53]	N = 94	mean 80, sd 8	Comorbidity	CCI	Health-related quality of life; influence of healthcare dependency measures on HRQoL or vice versa	Telemonitoring system: digital pen technology supported by hospital-based home care	Interventional (Pre-post study design)	Intervention	Enhancing quality of life
Ritt et al., 2017 [60]	N = 123	mean 82.4, sd 6.25	Frailty	Fried criteria; Frailty Index; Clinical Frailty Scale; Frailty index based on a comprehensive geriatric assessment assessment	Spatio-temporal gait parameters	Electronic walkway; shoe-mounted inertial sensor-based mobile gait analysis system.	Observational (Diagnostic accuracy study design)	Assessment	Detecting frailty satus from gait analysis
Sargent et al., 2020 [74]	N = 1453	mean 79, sd 0.54	Frailty	Fried criteria; MMSE score ≤ 23, TMT-A score ≥ 78, TMT-B score ≥ 106	Cognitive frailty: MMSE score ≤ 23, TMT-A score ≥ 78, TMT-B score ≥ 106; Physical frailty: ≥ 3 Fried criteria	Machine Learning - tree boosting approach model	Observational (Retrospective cohort study design - Predictive model)	Predicition	Studying biological mechanisms that relate physical frailty and cognitive impairment.
Schiltz et al., 2020 [75]	N = 6.617	≥ 65	Multimorbidity	Self-reported multimorbidity	30 day hospital readmission	Machine Learning - Random forest analysis, Classification and regression tree, Modified Poisson regression analysis, generalized estimating equation approach	Observational (Retrospective cohort study design - Predictive model)	Predicition	Studying IADL dependency as a predictor of hospital readmissions
Takahashi et al., 2012 [47]	N = 205	mean 80.3, sd 8.2	Multimorbidity	CIRS	Hospitalization and emergency department visits	Telemonitoring device	Interventional (Randomized controlled trial)	Intervention	Reducing hospitalizations and emergency department visits
Tomita et al., 2007 [48]	N = 78	mean 73.8, sd 4.7	Multimorbidity	CIRS	Functional status (ADL, IADL, MMSE, CHART)	Ambient assistive living: computer with internet access, X10-based smart home technology	Interventional (Randomized controlled trial)	Intervention	Studying feasibility and effectivenes of smart home technologies
Tsipouras et al., 2018 [61]	N = 73	mean 78.15, sd 5.5	Frailty	Fried criteria	Number and durantion of transitions	Bluetooth localization system: sensorobluetooth beacons, smartphone andMaschine Learning for frailty level assessment: Naïve Bayes classifier, k-Nearest Neighbour, Neural Networks, Decision Trees algorithm, Random Forests	Observational (Diagnostic accuracy study design)	Assessment	Correlation between indoor activities and frailty status
Violán et al., 2019 [78]	N = 916.619	mean 75.4, sd 7.4	Multimorbidity	> 1 of 60 chronic diseases	> 1of selected 60 chronic diseases; sociodemographics; number of invoiced drugs; use of health services	Machine Learning - fuzzy c-means clustering algorithm	Observational (Cross-sectional study design - Predictive model)	Predicition	Identifying multimorbidity patterns in the electronic health records
Volders et al., 2020 [50]	N = 585	mean 74.5, sd 6.4	Multimorbidity	Self-reported multimorbidity	Physical activity	ActiGraph GT3X-BT accelerometer	Interventional (Randomized controlled trial)	Intervention	Studying the effect of a computer-tailored phisical activity intervention

Number (N); standard deviation (sd); interquartile range (IQR); Charlson Co-morbidity Index (CCI); Minimental State Examination (MMSE); Trail Making Test – version A and B (TMT-A, TMT-B); Expanded Timed Up and Go test (ETUG); Activities of Daily Living (ADL); Instrumental Activities of Daily Living (IADL); Craig Handicap Assessment and Reporting Technique (CHART); Classification And Regression Tree salgoritm (CART); Information and Communication Technologies (ICT); Support Vector Machine (SVM); Linear Discriminant Analysis (LDA).

### Quality assessment

We chose the most appropriate quality assessment tool based on the records’ study design defined according to [41]. In particular, interventional randomized and non-randomized clinical trials were assessed using the Cochrane Collaboration’s Risk-of-Bias Tool [42]. Observational studies with diagnostic aims were assessed using the Quality Assessment of Diagnostic Accuracy Studies tool – second version (QUADAS-2) [43]. Observational studies using predictive models with prognostic purposes were assessed through the Quality In Prognosis Studies (QUIPS) tool [44]. See Supplementary materials for details of the scales used.

Six blinded researchers (A.G., P.D.T., C.T., S.C., A.GR., F.R.G.) in pairs evaluated the studies’ quality. Conflicts were solved by consensus of authors in each pair or by the involvement of a third author in case of discrepancies.

## Results

### Review selection

**Fig. 1:**
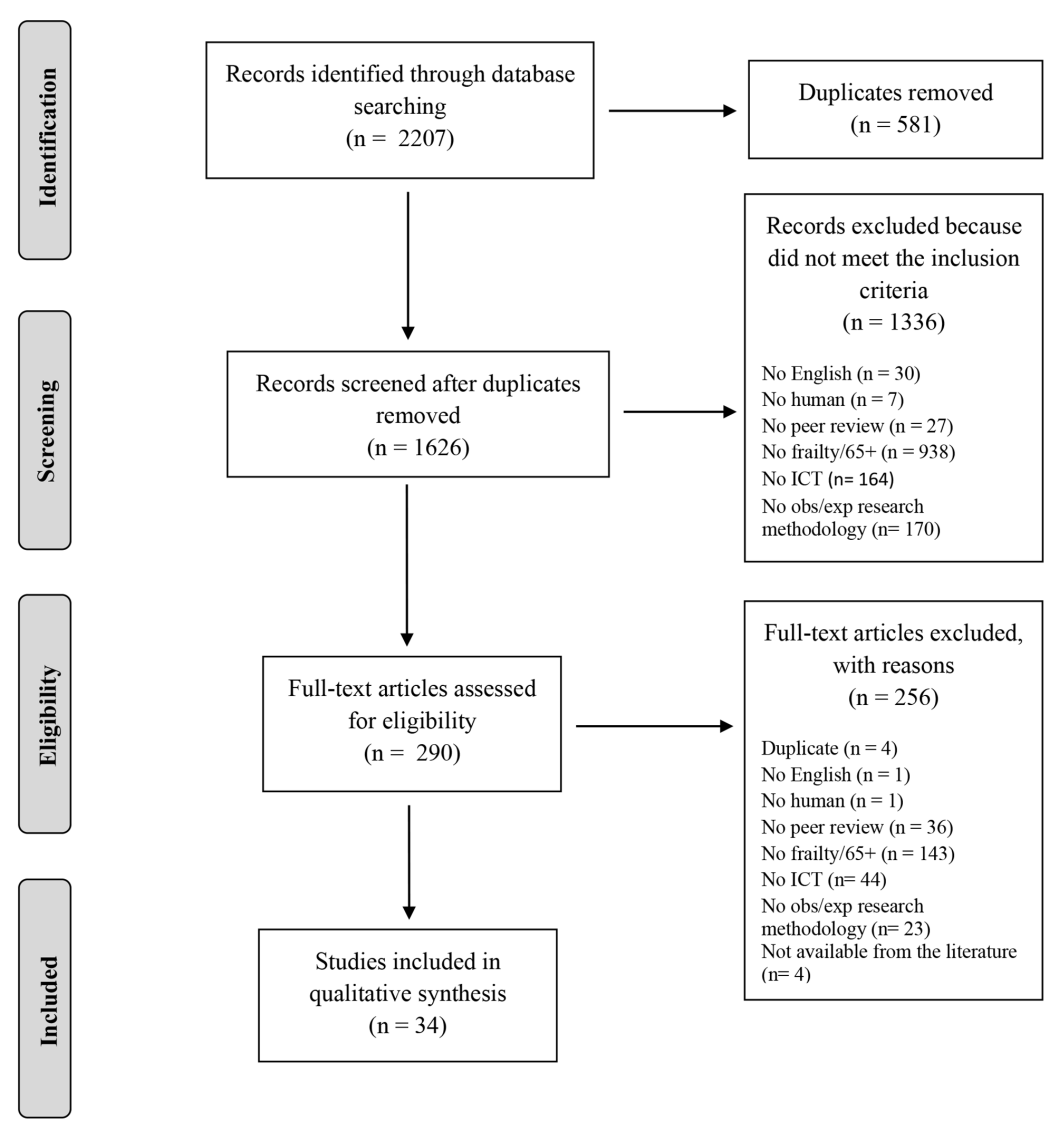
PRISMA Flowchart of the screening process

Based on our keywords we retrieved 2207 records. After removing duplicates, we screened the title and abstract of 1626 papers. According to our selection criteria and following the consensus on conflicts, 290 studies moved then to the full-text screening. Among the 290 full texts, 34 papers met our inclusion criteria and were included in the qualitative synthesis (Fig. 1).

### Quality assessment

**Fig. 2:**
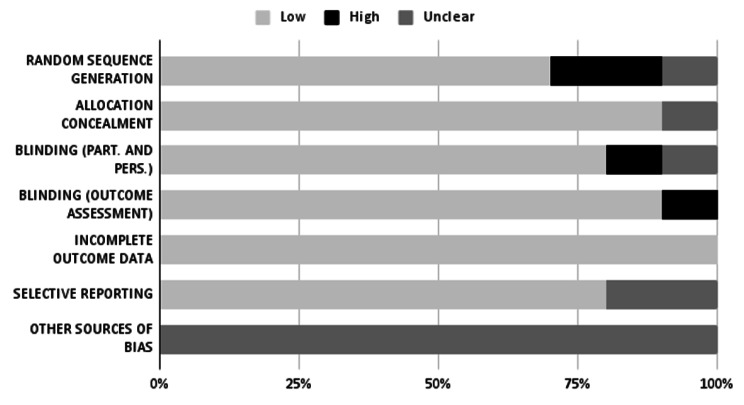
Risk of bias evaluation with Cochrane tool

**Fig. 3a:**
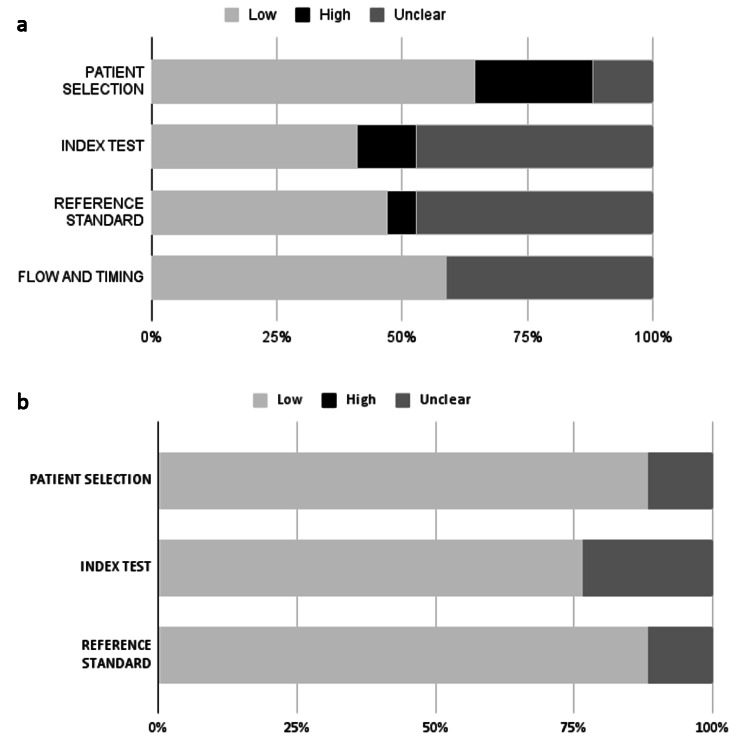
**(a)** Risk of bias evaluation with QUADAS-2 Risk tool. **(b)** (b)

**Fig. 4:**
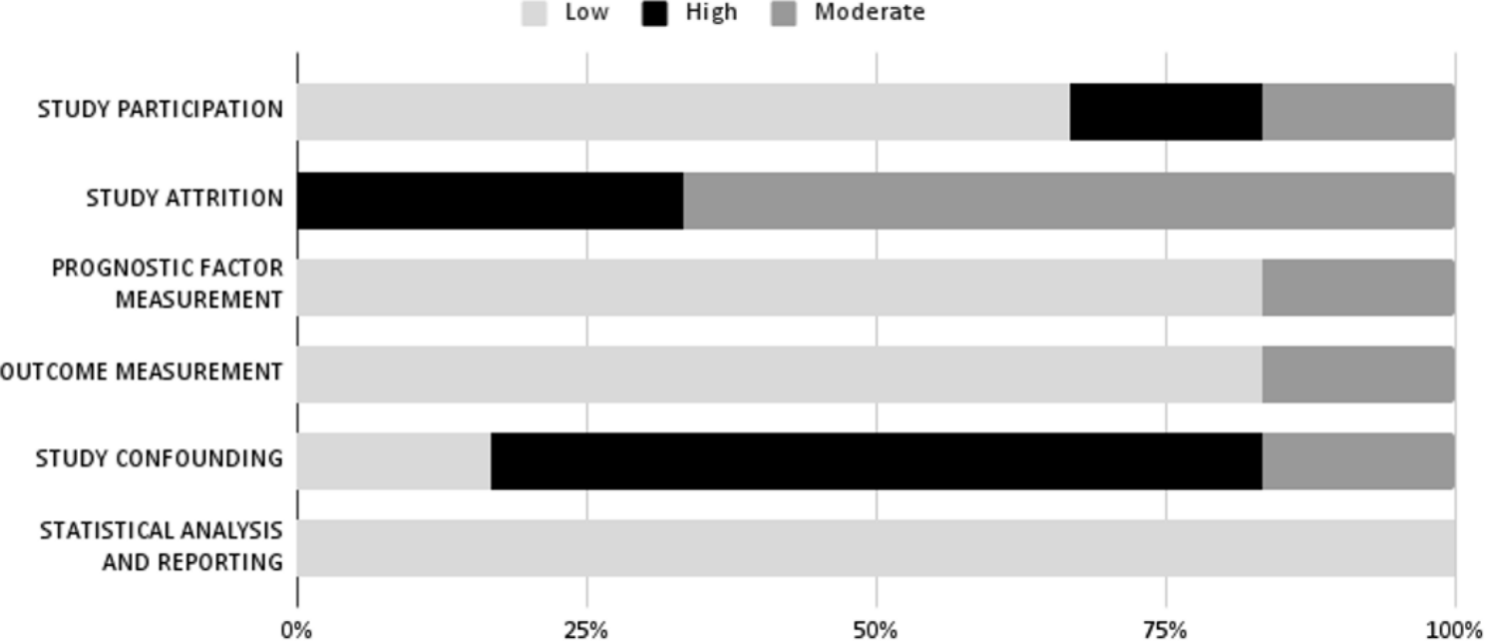
Risk of bias evaluation with QUIPS tool

The quality and the risk of bias assessment of the retrieved studies are summarized in Figs. 2, 3 and 4. Considering the Cochrane Collaboration’s Risk-of-Bias Tool (Fig. 2), we evaluated 10 papers out of 34. Among these, six were randomized control trials [45–50], two were non-randomized control trials [51, 52], one was a pre-post design [53] and one was a cross-over randomized control trial [54]. The analysis of the risk of bias across studies revealed a high average quality with only two studies exposed to selection bias [52, 53] and two studies exposed to performance bias [45, 49].

Based on the QUADAS-2 ratings (Fig. 3a), we assessed 15 [55–69] diagnostic accuracy studies, one retrospective cohort study [70], and one cross-sectional study [71]. The average quality of the included studies was intermediate to low as most of the analyzed items, in particular the tested index, the standard of reference, and timing of measurements, despite not totally at high risk of bias, were evaluated as unclear due to the lack of information in most of the retrieved papers. The item regarding the selection of patients was assessed as low risk by most of the papers, however, compared to the other items, it obtained the higher number of high risk ratings. Considering the evaluation of the applicability through QUADAS-2 (Fig. 3b), the quality of the retrieved observational studies was high.

We used the QUIPS tool to evaluate the quality of six observational studies describing a predictive model (Fig. 4). Among these, five papers were retrospective cohort studies [72–76] and one paper was a cross-sectional study [77]. The average quality was intermediate to low as the risk of attrition and confounding factors, as well as bias in patients’ section, were rated from moderate to high by most of the studies in this group.

### Research methodology

The majority of the selected papers (24/34) reported observational studies, whereas only ten out of thirty-four studies were interventional researches (Table 1). Below we report the main results organizing the selected studies according to these two major methodological approaches.

#### Observational studies

Twenty-four out of thirty-four papers were observational studies and involved 923,319 patients affected by multimorbidity [70, 75, 78], 90,096 affected by frailty [55–69, 72–74, 76] and 1,765 patients affected by geriatric syndromes [71, 77]. The diagnostic accuracy study design was the most represented study typology, with fifteen out of twenty-four studies [55–69], and the general aim of all these studies was to assess a diagnostic methodology devoted to detect frailty. The second most represented study typology was the retrospective cohort study design, with six out of twenty-four studies. The general objective of five of these studies was to elaborate a predictive model for frailty [72–74, 76] or multimorbidity [75], whereas the general aim of the last one was to study the mortality rate of patients suffering from multimorbidity [70]. The typology of the remaining three papers refered to cross-sectional study design. The general aim of two of these was to study prevalence of geriatric syndromes [71, 77] and the third study aimed to elaborate a predictive model for multimorbidity [78].

The twenty-four observational studies selected implemented different ICT technologies. Among the fifteen diagnostic accuracy studies, wearable sensors were the most common ICT technologies used and were mainly proposed to analyze postural and movement variables [55, 59–61, 67]. Four studies proposed to exploit widely diffuse technologies (smartphone or tablet) to analyze movement parameters during sit-to-stand tasks [64–66] or handwriting [63]. Two studies implemented virtual reality technologies, one to propose serious games [58] and the other to implement movement analysis [57]. The remaining four studies were based on different technologies: one study implemented a social robot to administer clinical questionnaires [62], another one assessed motion and walking parameters using commercial motion capture sensors like Kinect [68], a third one implemented a home monitoring platform with ambient sensors to analyze patient daily habits [69], and the last study used machine learning to identify frailty from administrative data [56]. Among the six retrospective cohort studies selected, five exploited different machine learning methods to develop predictive models for frailty [72–74, 76] or multimorbidity [75], whereas one study analyzed data from a telemonitoring experience to assess mortality according to multimorbidity and telemonitoring status [70]. The remaining three cross sectional studies implemented, in two cases, machine learning algorithms to generate predictive models for multimorbidity [78] or to detect patterns of medication combinations according to geriatric syndrome status [77], and in one case a mobile iPad application was used to study the prevalence of geriatric syndromes [71].

#### Interventional studies

Ten out thirty-four papers were interventional studies and involved 1912 patients affected by multimorbidity [46–49, 52], 170 affected by frailty [45, 50, 51, 54] and 53 with comorbidity [53].

Seven studies were randomized controlled trials, four devoted to study interventions for multimorbidity [46–49] and three for frailty [45, 50, 54]. The remaining three studies were two non-randomized trials, one for frailty [51] and one for multimorbidity [52], and one a pre-post study design which analyzes an intervention for comorbidity [53].

The seven randomized controlled trials selected used different ICT technologies. Three studies promoted physical activity (PA) or motivation toward PA through virtual reality [50], robot [54] or a web-based motivational program [49]; two studies analysed Ambient Assisted Living (AAL) interventions [45, 48], one study used telemonitoring [47] and another one a video consultation intervention [46]. The three further non-randomized trials proposed a smart-phone based PA program [51], a telemonitoring system [53] or an integrated care intervention [52].

## Discussion

The present review first aimed to systematically describe research designs implemented by studies about technologies applications to clinical assessment and treatment of aging-related syndromes. Overall, the results showed a clear imbalance toward a more represented amount of observational studies compared to interventional ones. This result reflects the well-known limits of applying only standard Randomized Clinical Trials (RCTs) in the research field of technology-based interventions for rehabilitation purposes [79]. Moreover, the quality assessment revealed that interventional studies were of higher quality, whereas observational studies were mainly of intermediate to low quality. Taken together, these findings may suggest that the field is still seminal as emerged in a previous review [4].

Our second aim was to propose a step model to standardize studies’ methods and improve the research quality in this field. This is in line with the actions of the European Network for Health Technology Assessment. This network on technology research recommends: a clear assessment of previous studies’ results; the disclosure of the rationale for using technology; the clinical indication of the population, the kind of intervention and comparators; the evidence about safety and effectiveness; the definition of study design (see https://www.eunethta.eu/methodology-guidelines/). Based on these recommendations, we hypothesize that one possible interpretation of our results could be the absence of a strong frame of reference describing all the steps useful to obtain a technology of good quality to be used for diagnostic or interventional purposes in a real-world setting. Considering the field of new drugs development, suggestions on how to formulate a canonized frame helping to overcome the emerged limitations could be found. Indeed, we propose that the development of a new technology to be used for diagnostic or interventional purposes has to pass several steps similar to those of the process to develop a new drug, as shown in Table 2.

**Table 2:** 5-step Model

Drug Development phases	Technology Development phases – 5 step Model
Preclinical studies (i.e., aninal testing)	Development of a new technology or adaptation of an existing one
Phase 1, proof of safety (i.e., study of drug pharmacokinetic and pharmacodynamics)	Target population analysis; technology usability, acceptability and safety
Phase 2, proof of principle (i.e., preliminary controlled studies on drug efficacy)	Clinical research, RCTs on small selected groups*
Phase 3, pivotal studies (i.e., large effectiveness studies on heterogeneous populations)	Clinical research, pragmatic design studies on large groups in a real-world setting with effectiveness and cost-efficacy analyses*
Phase 4, post-Market monitoring	Review from a recognized Institution and Post-Market monitoring

*If the technology is intended for diagnostic purposes, the “Clinical research” phases would comprise observational studies with the

aim to analyze validity, reliability, sensitivity, and specificity first in small selected groups and then in large groups in a real-world

setting

In our model, the two phases of the “Clinical research” step represent the attempt to exploit the power of “Efficacy” studies, which are intended to assess the performance of an intervention under ideal circumstances, as well as “Effectiveness” studies, which are intended to produce evidence of therapeutic effectiveness in real-world practice settings [80, 81]. Usually “Efficacy” studies are the classical RCTs that, even though represent the gold standard for evaluating the efficacy of an intervention, require highly controlled conditions to avoid biases and confounding factors [81]. On the other hand, “Effectiveness” studies are retrospective or prospective real-world observational studies that, by using a less strict methodology and examining interventions under circumstances closer to real-world practice, lead to complementary evidence to that provided by RCTs, even if they are more prone to several sources of bias and risk of uncontrolled confounders [80].

Considering these characteristics and the concerns of limiting the research on technologies for disabilities only to RCTs [79], we think that both these types of studies should contribute to the research field of technologies applications for the diagnosis and intervention of age-related pathologies. Indeed, the results of this review, coherently with previous works [4, 5], show that studies in which technological solutions are tested in samples of frail, comorbid, and multimorbid old patients frequently describe technical aspects of technologies through laboratory experimentations, with only empirical applications on pilot samples. In other cases, they are large but methodologically poor observational studies aiming at improving the assessment or at providing predictive models. Sometimes, they are clinical trials that, despite the good quality, lack usability and acceptability considerations and are underpowered to generalize the results or to run cost-effectiveness analyses. Therefore, we first suggest that systematizing “Efficacy” and “Effectiveness” study designs in the “Clinical research” phase of our model could enhance the methodological rigor of randomized trials and observational studies, both conducted on small selected samples as well as on heterogeneous and large groups of patients. Following the methodological requirements of “Efficacy” and “Effectiveness” research designs, RCTs could indeed rightly test technologies efficacy, while observational studies could strictly explore aging-related syndromes prevalence, technologies applications’ validity, reliability, sensitivity, and their role in predicting long-term outcomes of chronic conditions. Second, considering the proposed model as a whole, we are confident that applying such a rigorous framework could help scholars to dialogue with clinicians, to effectively investigate technologies’ usability, acceptability, and safety based on clinical population characteristics and strata. This, in turn, could prepare the stage for well-designed clinical studies that could provide solid results, even regarding cost-effectiveness analysis, to be used for revision and approval by a recognized Institution and finally for post-market monitoring of long-term effects and large-scale use.

In conclusion, the results of the present systematic review seem to suggest that research in the field of the development and use of technological tools for aging-related syndromes is, at the moment, mostly oriented toward observational studies devoted to diagnostic tools to be used in the assessment of geriatric conditions (e.g. frailty). The field suffers from some limitations related to the research quality and poor attention to interventional studies of efficacy and effectiveness. We propose that a structured and shared methodological approach, like that followed in pharmaceutical studies, could help the field to increase the research quality and more adequately respond to the needs of patients and their caregivers.

## Electronic supplementary material

Below is the link to the electronic supplementary material.

Supplementary Material 1

Supplementary Material 2

## Data Availability

All data generated or analysed during this study are included in this published article [and its supplementary information files].
