# Correction to: Technologies for frailty, comorbidity, and multimorbidity in older adults: a systematic review of research designs

**DOI:** 10.1186/s12874-023-01996-4

**Published:** 2023-08-01

**Authors:** Alessia Gallucci, Pietro D. Trimarchi, Cosimo Tuena, Silvia Cavedoni, Elisa Pedroli, Francesca Romana Greco, Antonio Greco, Carlo Abbate, Fabrizia Lattanzio, Marco Stramba-Badiale, Fabrizio Giunco

**Affiliations:** 1 IRCCS Fondazione Don Carlo Gnocchi, Milan, Italy; 2 Applied Technology for Neuro‑Psychology Lab, IRCCS Istituto Auxologico Italiano, Milan, Italy; 3 Faculty of Psychology, University of eCampus, Novedrate, Italy; 4 Geriatric Unit, Department of Medical Sciences, IRCCS ‘‘Casa Sollievo della Sofferenza’’, San Giovanni Rotondo, Italy; 5 Scientific Direction, IRCCS INRCA, Ancona, Italy; 6 Department of Geriatrics and Cardiovascular Medicine, IRCCS Istituto Auxologico Italiano, Milan, Italy

**Correction: BMC Medical Research Methodology 23, 166 (2023)**

10.1186/s12874-023-01971-z

Following the publication of the original article [1], the authors requested to correct Dr. Cosimo Tuena‘s affiliation to “Applied Technology for Neuro‑Psychology Lab, IRCCS Istituto Auxologico Italiano, Milan, Italy”.

The original article [1] has been updated.
