# Supplementary material for: Technologies for frailty, comorbidity, and multimorbidity in older adults: a systematic review of research designs
Source: BMC Med Res Methodol. 2023 Jul 11;23:166. doi: 10.1186/s12874-023-01971-z (PMC10334509; doi:10.1186/s12874-023-01971-z)
Supplement: Supplementary file 1 — Supplementary Material 1 [file 12874_2023_1971_MOESM1_ESM.docx]

**Technologies’ keywords**

1. ICT: “robot”, “information and communication technology”, “assistive technologies”, “social network”, “smart homes”, “ambient assisted living”, “telemedicine”, “telehealth”, “telemonitoring”, “telecare”, “telerehabilitation”, “teleassessment”, “medication optimization”, “technology-based”.
2. Machine learning: “machine learning”, “artificial intelligence”.
3. Virtual reality: “computer”, “smartphone”, “iphone”, “tablet”, “ipad”, “projectors”, “CAVE”, “visor”, “head-mounted display”, “simulator”, “virtual”.

**Quality assessment tools**

The Cochrane Collaboration’s Risk-of-Bias Tool allows to assess as “high risk”, “low risk”, “unclear risk” or “risk not applicable” the following sources of bias: a) selection bias referring to randomization procedures and allocation concealment; b) performance bias regarding blinding of participants and research staff; c) detection bias concerning blinding of assessors and data handlers/analysts; d) attrition bias referring to drop-outs and missing data; e) reporting bias concerning systematic errors in reporting study outcomes.

The QUADAS-2 encompasses two levels of evaluation: a) the “study level” assessed across four domains: patients selection, diagnostic procedures of studying, diagnostic standard of reference, timing of measurements; b) the “applicability level” regarding whether the four domains fit the research question of the systematic review. At both levels, items in each domain are evaluated as “high risk”, “low risk” or “unclear risk”.

The QUIPS considers the following domains as possible sources of bias: a) study participation; b) study attrition; c) prognostic factor measurement; d) outcome measurement; e) study confounding; f) statistical analysis and reporting. Each item is assessed as “yes” for low risk of bias and “no” for high risk of bias.

**Appendix A – Descriptors of search**

| Searchengine | Searchalgorithm |
| --- | --- |
|  |  |
| PubMed  (September 2020) | ***Series 1:*** ((("aging"[MeSH Terms] OR "aging"[All Fields]) AND ("frailty"[MeSH Terms] OR "frailty"[All Fields])) AND ("multimorbidity"[MeSH Terms] OR "multimorbidity"[All Fields] AND ("elder*"[MeSH Terms] OR "elder*"[All Fields]))) AND ("Integr Cancer Ther"[Journal] AND "**ict**"[All Fields])  ***Series 2:*** ((("aging"[MeSH Terms] OR "aging"[All Fields]) AND ("frailty"[MeSH Terms] OR "frailty"[All Fields])) AND ("multimorbidity"[MeSH Terms] OR "multimorbidity"[All Fields] AND ("elder*"[MeSHTerms] OR "elder*"[All Fields]))) AND ("Integr Cancer Ther"[Journal] AND ("**case management**"[MeSH Terms] OR ("case"[All Fields] AND "management"[All Fields]) OR "case management"[All Fields])) OR (care[All Fields] AND ("organization and administration"[MeSH Terms] OR ("organization"[All Fields] AND "administration"[All Fields]) OR "organization and administration"[All Fields] OR "management"[All Fields] OR "disease management"[MeSH Terms] OR ("disease"[All Fields] AND "management"[All Fields]) OR "disease management"[All Fields]))  ***Series 3:*** ((("aging"[MeSH Terms] OR "aging"[All Fields]) AND ("frailty"[MeSH Terms] OR "frailty"[All Fields])) AND ("multimorbidity"[MeSH Terms] OR "multimorbidity"[All Fields] AND ("elder*"[MeSH Terms] OR "elder*"[All Fields]))) AND ("Integr Cancer Ther"[Journal] AND "**ict**"[All Fields])AND ("case management"[MeSH Terms] OR ("case"[All Fields] AND "management"[All Fields]) OR "case management"[All Fields])) OR (care[All Fields] AND ("organization and administration"[MeSH Terms] OR ("organization"[All Fields] AND "administration"[All Fields]) OR "organization and administration"[All Fields] OR "management"[All Fields] OR "disease management"[MeSH Terms] OR ("disease"[All Fields] AND "management"[All Fields]) OR "disease management"[All Fields]))  ***Series 4:*** ((("aging"[MeSH Terms] OR "aging"[All Fields]) AND ("frailty"[MeSH Terms] OR "frailty"[All Fields])) AND ("multimorbidity"[MeSH Terms] OR "multimorbidity"[All Fields] AND ("elder*"[MeSH Terms] OR "elder*"[All Fields]))) AND ("Integr Cancer Ther"[Journal] AND "**ict**"[All Fields])AND ("delivery of health care"[MeSH Terms] OR ("delivery"[All Fields] AND "health"[All Fields] AND "care"[All Fields]) OR "delivery of health care"[All Fields] OR ("health"[All Fields] AND "care"[All Fields]) OR "health care"[All Fields]) |
| Web of Science  (September 2020) | *Series 1:* (ALL=(Aging AND Frailty AND Multimorbidity AND Elder* AND ICT)) AND LANGUAGE: (English) AND DOCUMENT TYPES: (Review)*Series 2:* (ALL=(Aging AND Frailty AND Multimorbidity AND Elder* AND (case management OR care management)) AND LANGUAGE: (English) AND DOCUMENT TYPES: (Review)*Series 3:* (ALL=(Aging AND Frailty AND Multimorbidity AND Elder* AND ICT) AND (case management OR care management)) AND LANGUAGE: (English) AND DOCUMENT TYPES: (Review)*Series 4:* (ALL=(Aging AND Frailty AND Multimorbidity AND Elder* AND ICT) AND health care) AND LANGUAGE: (English) AND DOCUMENT TYPES: (Review) |
| EMBASE  (September 2020) | **ICT arm:** (frail*:ti,ab,kw OR multimorbility:ti,ab,kw OR 'comorbidity'/mj OR comorbidity:ti,ab,kw) AND ('aging'/mj OR aging:ti,ab,kw OR elder*:ti,ab,kw) AND (robot*:ti,ab,kw OR 'information and communicationtechnology'/mj OR 'information and communicationtechnology':ti,ab,kw OR 'assistivetechnolog*:ti,ab,kw' OR 'social network*':ti,ab,kw OR 'smarthom*':ti,ab,kw OR 'ambient assisted living'/mj OR 'ambient assisted living':ti,ab,kw OR 'telemedicine'/mj OR telemedicine:ti,ab,kw OR 'telehealth'/mj OR telehealth:ti,ab,kw OR 'telemonitoring'/mj OR telemonitoring:ti,ab,kw OR 'telecare'/mj OR telecare:ti,ab,kw OR 'telerehabilitation'/mj OR telerehabilitation:ti,ab,kw OR teleassessment:ti,ab,kw OR 'medicationoptimization':ti,ab,kw OR 'technology-based':ti,ab,kw)  **Machine learning arm:** (frail*:ti,ab,kw OR multimorbility:ti,ab,kw OR 'comorbidity'/mj OR comorbidity:ti,ab,kw) AND ('aging'/mj OR aging:ti,ab,kw OR elder*:ti,ab,kw) AND ('machine learning'/mj OR 'machine learning':ti,ab,kw OR 'artificial intelligence'/mj OR 'artificial intelligence':ti,ab,kw) Virtual reality arm: (frail*:ti,ab,kw OR multimorbility:ti,ab,kw OR 'comorbidity'/mj OR comorbidity:ti,ab,kw) AND ('aging'/mj OR aging:ti,ab,kw OR elder*:ti,ab,kw) AND ('computer'/mj OR computer:ti,ab,kw OR 'smartphone'/mj OR smartphone:ti,ab,kw OR 'iphone'/mj OR iphone:ti,ab,kw OR 'tablet'/mj OR tablet:ti,ab,kw OR 'ipad'/mj OR ipad:ti,ab,kw OR projector*:ti,ab,kw OR 'cave'/mj OR cave:ti,ab,kw OR visor:ti,ab,kw OR 'head-mounted display'/mj OR 'head-mounted display':ti,ab,kw OR 'simulator'/mj OR simulator:ti,ab,kw OR virtual:ti,ab,kw) |

**Appendix B – PRISMA checklist**

| **Section/topic** | **#** | **Checklist item** | **Reported on page #** |
| --- | --- | --- | --- |
| **TITLE** | | |  |
| Title | 1 | Identify the report as a systematic review, meta-analysis, or both. | Page 1 |
| **ABSTRACT** | | |  |
| Structured summary | 2 | Provide a structured summary including, as applicable: background; objectives; data sources; study eligibility criteria, participants, and interventions; study appraisal and synthesis methods; results; limitations; conclusions and implications of key findings; systematic review registration number. | Page 3 |
| **INTRODUCTION** | | |  |
| Rationale | 3 | Describe the rationale for the review in the context of what is already known. | Page 4-6 |
| Objectives | 4 | Provide an explicit statement of questions being addressed with reference to participants, interventions, comparisons, outcomes, and study design (PICOS). | Page 6 |
| **METHODS** | | |  |
| Protocol and registration | 5 | Indicate if a review protocol exists, if and where it can be accessed (e.g., Web address), and, if available, provide registration information including registration number. | Page 7 |
| Eligibility criteria | 6 | Specify study characteristics (e.g., PICOS, length of follow-up) and report characteristics (e.g., years considered, language, publication status) used as criteria for eligibility, giving rationale. | Page 7-8 |
| Information sources | 7 | Describe all information sources (e.g., databases with dates of coverage, contact with study authors to identify additional studies) in the search and date last searched. | Page 7 and Appendix A |
| Search | 8 | Present full electronic search strategy for at least one database, including any limits used, such that it could be repeated. | Appendix A and Supplementary material |
| Study selection | 9 | State the process for selecting studies (i.e., screening, eligibility, included in systematic review, and, if applicable, included in the meta-analysis). | Page 7, Page 9 and Figure 1 |
| Data collection process | 10 | Describe method of data extraction from reports (e.g., piloted forms, independently, in duplicate) and any processes for obtaining and confirming data from investigators. | Page 8 and Table1 |
| Data items | 11 | List and define all variables for which data were sought (e.g., PICOS, funding sources) and any assumptions and simplifications made. | Page 8, Table 1 and Supplementary material |
| Risk of bias in individual studies | 12 | Describe methods used for assessing risk of bias of individual studies (including specification of whether this was done at the study or outcome level), and how this information is to be used in any data synthesis. | Page 8 and Supplementary material |
| Summary measures | 13 | State the principal summary measures (e.g., risk ratio, difference in means). | Not applicable |
| Synthesis of results | 14 | Describe the methods of handling data and combining results of studies, if done, including measures of consistency (e.g., I^2^) for each meta-analysis. | Not applicable |

| Risk of bias across studies | 15 | Specify any assessment of risk of bias that may affect the cumulative evidence (e.g., publication bias, selective reporting within studies). | Page 8 and Supplementary material |
| --- | --- | --- | --- |
| Additional analyses | 16 | Describe methods of additional analyses (e.g., sensitivity or subgroup analyses, meta-regression), if done, indicating which were pre-specified. | Not applicable |
| **RESULTS** | | |  |
| Study selection | 17 | Give numbers of studies screened, assessed for eligibility, and included in the review, with reasons for exclusions at each stage, ideally with a flow diagram. | Page 9 and Figure 1 |
| Study characteristics | 18 | For each study, present characteristics for which data were extracted (e.g., study size, PICOS, follow-up period) and provide the citations. | Page 10-12 and Table 1 |
| Risk of bias within studies | 19 | Present data on risk of bias of eachstudy and, ifavailable, anyoutcomelevelassessment (see item 12). | Page 9-10 and Table 2-4 |
| Results of individual studies | 20 | For all outcomes considered (benefits or harms), present, for each study: (a) simple summary data for each intervention group (b) effect estimates and confidence intervals, ideally with a forest plot. | Not applicable |
| Synthesis of results | 21 | Present results of each meta-analysis done, including confidence intervals and measures of consistency. | Not applicable |
| Risk of bias across studies | 22 | Presentresults of anyassessment of risk of biasacrossstudies (see Item 15). | Page 9-10 and Table 2-4 |
| Additional analysis | 23 | Give results of additional analyses, if done (e.g., sensitivity or subgroup analyses, meta-regression [see Item 16]). | Not applicable |
| **DISCUSSION** | | |  |
| Summary of evidence | 24 | Summarize the main findings including the strength of evidence for each main outcome; consider their relevance to keygroups (e.g., healthcare providers, users, and policy makers). | Page 13-15 and Table 2 |
| Limitations | 25 | Discuss limitations at study and outcome level (e.g., risk of bias), and at review-level (e.g., incomplete retrieval of identified research, reporting bias). | Page 13-15 |
| Conclusions | 26 | Provide a general interpretation of the results in the context of other evidence, and implications for future research. | Page 15 |
| **FUNDING** | | |  |
| Funding | 27 | Describe sources of funding for the systematic review and other support (e.g., supply of data); role of funders for the systematic review. | Page 2 |
